# On the Injection of Stimulants into the Veins

**Published:** 1849-11

**Authors:** J. M. Steiner

**Affiliations:** Assistant Surgeon U. S. Army; Fort Graham, Texas


*On the Injection of Stimulants into the Veins.* By J. M. Steiner, M. D., Assistant Surgeon U. S. Army. (Communicated in a letter to one of the Editors.

With the permission and assistance of Lieutenant Fowler Hamilton, 2d Dragoons commanding this Post, I instituted a series of experiments upon four public horses, condemned to be shot, with the view of discovering what symptoms would result from the injection of stimulants into the general circulation.

Aug. 21.—1st. A large horse, affected with an eruptive disease of the skin, termed farcy, was secured, and an incision made into the right external jugular vein. By means of a syringe, one drachm of pure alcohol was thrown into the vessel, and its effects carefully noted. In the course of a minute the eyelids became half closed, and the animal appeared as if under the influence of a narcotic. The same quantity of alcohol was injected at intervals of two minutes, until the whole amounted to six drachms. The pulse increased in strength and frequency, and an abundant perspiration exuded in large drops upon the right hip and thigh.

2d. This horse was affected with the Spanish fever. Three drachms of alcohol were injected into the jugular vein, without producing any other effect than a slight acceleration of the pulse. Five minutes after, one and a half drachms of sulphuric ether were thrown into the same orifice, but without giving rise to any additional symptoms.

3d. A horse with the farcy. Two ounces and a half of alcohol undiluted, were injected into the jugular vein. I passed the tips of my fingers along the course of the vein as far as the base of the neck, and could distinctly hear the gurgling of the *air* which was thrown into the vessel along with the alcohol.* In less than half a minute, the animal trembled violently, staggered and fell; regained his feet and fell again. Upon applying the hand, the heart could be felt throbbing violently against the chest, and its pulsations distinctly heard without placing the ear in contact with the surface. He laid quiet for five minutes, and from the appearance of his eyes, and the notice he took of the hand, when waved before them, seemed perfectly sensible. At the expiration of the time mentioned, he was whipped to his feet, and went to grazing.

4th. This horse was stifled. Two ounces of aqua ammoniæ, diluted with an equal quantity of water, were injected into the jugular. In fifteen seconds the upper lip and nostrils became vio-

* In this case the instantaneous effects usually resulting from the injection of air were not produced. Some animals may receive enormous quantities of air into the veins without causing death. Magendie relates that on one occasion he introduced with a syringe, with his whole strength, and as rapidly as possible, from twenty to twenty-four pints into the veins of an old horse, without causing immediate death, though he ultimately sank. After death the whole circulatory system was found filled with air, and even the lymphatic system was distended with yellowish lymph, slightly mixed with air.

lently convulsed, when, staggering forward, he fell upon his knees, but instantly regained his feet. For a few moments he looked frightened and astonished, and then went to cropping the grass.

I have since (August 23d) repeated the experiments upon three of the same horses. Three ounces of aqua ammoniæ, diluted with water, were injected into the jugular of the first, four ounces of the oil of turpentine, undiluted, into that of the second,* and eight ounces of whiskey into that of the 3d. At the expiration of a minute, the first horse fell and was thrown into convulsions, lasting for a half a minute. In the course of five minutes he was whipped to his feet, and moved off with great difficulty. The horse that had received the turpentine, did not fall, but reeled and staggered for ten minutes, and for several hours after walked as if blind and drunk. The third horse experimented upon with the whiskey, remained firm and stood upon his feet, without any untoward symptoms supervening. In the three instances, an abundant perspiration broke out over the whole body before the expiration of fifteen minutes.

It is the opinion of some physiologists, that alcohol, or any other substance in its " active state,"† cannot be injected into the circulation of man without producing instantaneous death. Some aver, that as soon as the stimulant reaches the heart, that organ becomes paralysed. Such is the inference, I have been informed, deduced from experiments made upon some of the lower animals. The fairness of such an inference seems to admit of some question, and when we consider the vital importance of this subject, a too ready credence should not be yielded to conclusions not based upon actual and carefully repeated experiments. Why is it that alcohol, when injected into the circulation at sufficient distance from the heart, to become mixed with and diffused through the blood, should have the effect of paralysing that organ, while that absorbed from the stomach is followed by no such result? Can it be, that the contents of the stomach essentially modify the qualities of the alcohol, or can it meet with any change in passing through the liver beyond that described by Magendie, who believes that the passage of foreign fluids into the animal economy,

* The horse that received the turpentine, died of hæmaturia on the 25th.

† Chapman's Elements of Therapeutics, p. 47.

through the innumerable small vessels of the liver, has the effect of mixing them more intimately with the blood, and, as it were, diluting them with a large quantity of the fluid, so that their chemical nature becomes somewhat altered? Or, what is more probable, do not the instantaneously fatal results of the infusion, depend rather upon the incautious and rapid manner in which such substances are injected? If fifteen grains of bile be forced suddenly into the crural vein of a dog, death will result in a few moments; but if it be *slowly* injected, it does not produce any sensible injury. Magendie's own experiments, as detailed by himself, and those of Blundell and Lepelletier, as described by Dunglison,* as well as the frequent saline injections in cholera, prove that the fatal results follow, not so much from the presence of the foreign substance in the blood, as from the incautious mode of proceeding.

If the hypothesis of those who maintain this *doctrine of paralysis* be true, some such change as that above mentioned must take place; and if so, it would prove both instructive and interesting, at least to some of us in the army, to know in what it consists. Had it not been for the prevalence of this hypothesis, I am inclined to believe, that numbers of the wounded in the late war with Mexico might have been saved by the injection of stimulants into the circulation, at a point as far as practicable from its center. The major part of the wounded struck by round shot and shell, died without the surgeon being able to bring about reaction. Large quantities of stimulants, brandy, ether, ammonia, &c., were freely administered, but after remaining in the stomach a few minutes, were thrown up, and when retained, were not absorbed. In these cases, could the stimulants have been injected into the circulation, *reaction* might frequently have resulted, and many lives in consequence perhaps have been preserved.

*Fort Graham, Texas, Aug.*, 1849.

* Human Physiology, Vol. I. p. 621.

